# Cross-cultural adaptation and psychometric assessment of the statement format Decisional Conflict Scale for Mandarin version

**DOI:** 10.1186/s12913-019-4717-6

**Published:** 2019-11-21

**Authors:** Cui Lu, Wei Mu, Ying-hui Jin, Yue-xian Shi, Ge Li, Yan Li, Fei Han, Tian Xia

**Affiliations:** 1 Emergency Department, TEDA Hospital, No.65, Third Road, Economic and Technological Development Zone, Tianjin, 300457 China; 2 Department of Clinical Pharmacology, Second Affiliated Hospital of Tianjin University of Traditional Chinese Medicine, No. 69, Zengchan Road, Hebei District, Tianjin, 300250 China; 3 Center for Evidence-Based and Translational Medicine, Zhongnan Hospital of Wuhan University, Center for Evidence-Based and Translational Medicine, Wuhan University, No.169, Donghu Road, Wuchang District, Wuhan City, 430071 Hubei Province China; 4 School of Nursing, Peking University, No.38 Xueyuan Road, Haidian District, Beijing, 100191 China; 5 Public Health Department, Tianjin University of Traditional Chinese Medicine, No.10 Boyanghu Road, Jinghai District, Tianjin, 301617 China; 6 Nursing school, Tianjin University of Traditional Chinese Medicine, No.10 Boyanghu Road, Jinghai District, Tianjin, 301617 China; 7 Emergency Department, Xuan Wu Traditional Chinese Medicine Hospital, No.8 Wanmingjia Road, Xicheng District, Beijing, 10000 China; 8 Center for Reproductive Medicine, First Teaching Hospital of Tianjin University of Traditional Chinese Medicine, No. 88 Changling Road, Xiqing District, Tianjin, 300381 China

**Keywords:** Health decision making, Validation studies, Reliability, Patient

## Abstract

**Background:**

The statement format of the Decisional Conflict Scale (sf-DCS) is designed and widely used to assess patients’ state of uncertainty during health related decision making. As yet no Mandarin version of the sf-DCS has been produced. This study aims to produce the first Mandarin version of the sf-DCS and test its validity and reliability in mainland China.

**Methods:**

The translation and cross-cultural adaptation of the original English version of the sf-DCS into Mandarin was carried out in accordance with previously published guidelines. The psychometric properties of sf-DCS were assessed in two hypothesized decision-making contexts through online surveys.

**Results:**

In the online survey designed to test scale validity and reliability, 437 people responded to the influenza immunization survey and 238 responded to the breast cancer screening survey. The results confirm that the Mandarin version of sf-DCS has good criteria validity and the exploratory factor analysis suggested a fitted revised five factors model by removing three items. Respondents who were “unsure” about their decisions/intentions, had read less information, and reported lower self-perceived prior knowledge level scored higher on sf-DCS. The Cronbach’s alpha for the sf-DCS total score was 0.963 and that for each subscale ranged from 0.784 to 0.937 in both decision making contexts, and the test-retest correlation coefficient was 0.528.

**Conclusions:**

The Mandarin version of sf-DCS has good criteria validity and its internal consistency is satisfactory. Our analysis suggests a refinement of the original sf-DCS’s factor structure is needed.

## Background

The Decisional Conflict Scale (DCS) was first developed in 1995 for measuring perceptions of uncertainties in the study of patient’s personal healthcare decision-making process [1]. Over the past two decades, it has been widely used as a measurement tool to assess the effect of decision support interventions, especially Patient Decision Aids (PDAs) [2–4]. PDAs are an important approach to Shared Decision Making (SDM) [5]. With growing recognition of SDM and patient involvement in medical decision making [2], personal health-related decision making could frequently pose a challenge for patients starting to take their own roles in healthcare. Therefore, to facilitate the patient’s safe self-stewardship in the face of uncertainty, it is very important to assess personal perceptions of uncertainty in choosing options and modifiable factors contributing to uncertainty such as feeling uninformed or unclear about personal values and unsupported in decision making [1], which is the precise purpose of the DCS.

According to Professor O’Connor, the developer of the DCS, the conceptual framework guiding its development was derived from the construct of decisional conflict, which was defined as a state of uncertainty about the course that has to be taken [1]. This state is likely when making choices involving uncertain outcomes because it is likely to lead the person to choose uncertainty [1, 6]. Given the multiple options available in health care decision making and the fact that medical science is based on probabilities, both patients and healthcare providers must weigh the risks of harm against the expected benefits of all options together using the best evidence available to make preference-sensitive decisions. As a result patients can be caught in the state of decisional conflict during decision making. Individuals experiencing decisional conflict may express the need to make value tradeoffs in selecting a course of action, regret over the positive aspects of rejected options and vacillation between choices, or suffer delayed decision making, self-focusing, signs of stress or tension, or undergo questioning of personal values and beliefs during decision making [7]. Therefore, the DCS is based on a fairly practical conceptual framework and clear manifestations.

To date, there are 4 versions of the DCS: one for clinical practice (the SURE test version) and three for research including a statement format, a question format and a low literacy format. The statement format of DCS (sf-DCS) has been most widely used to evaluate PDAs across different cultures, populations and contexts of medical decision making [2–4], and is popular among respondents. The sf-DCS has also been translated into many languages, in order to make it possible to compare results across different language contexts including versions in Dutch [8], French [9], Japanese [10] and German [11]. Currently there is a lack of, a tested, validated and reliable Mandarin version of the sf-DCS for use in mainland China. So there is a need to produce a Mandarin version to allow the relevant research results based on the population in mainland China to contribute to international cross-cultural studies in the future. This study aims to produce the first Mandarin version of the sf-DCS following the cross-cultural adaptation process stipulated in established guidelines and assess the psychometric properties of the scale.

## Methods

### The sf-DCS

The sf-DCS encompasses five subscales (uncertainty, feeling informed, clarity related to personal values, feeling supported, and effective decision making) involving 16 items that use a 5-point Likert-type response categories (i.e., strongly agree, agree, neither agree nor disagree, disagree, and strongly disagree) for scoring. Decisional conflict was calculated from calculating the total scores obtained on these 16 items. Items are given a score value of: 0 = “strongly agree”; 1 = “agree”; 2= “neither agree nor disagree”; 3 = “disagree”; 4 = “strongly disagree”. The total score and the score for each subscale are calculated according to the DCS user’s manual. The total score ranges from 0 to 64, and is typically converted to the 0–100 scale with 100 scores indicating extremely great decisional conflict. A copy of the original tool has been attached in the Supplementary information as Additional file 1.

### Translation and cross-cultural adaptation of the sf-DCS

The translation and cross-cultural adaptation of the original English version of the sf-DCS into Mandarin was carried out in accordance with previously published guidelines [12].

#### Translation and synthesis

Two native mainland Chinese speakers (T-1, T-2) with different job profiles (an associate professor and a senior nurse) independently translated the scale into Mandarin. The senior nurse was familiar with the concepts and content of the sf-DCS and the associate professor was neither aware nor informed of the concepts. The different backgrounds of the two translators and discussions to resolve discrepancies assured consistency with the original text in terms of both content and wording. An assistant researcher with a master’s degree in English translation was invited together with the two translators to compare the two translations. The two translations were compared with each another and with the original English version. After discussing any discrepancies that had arisen, a consensus was reached and the three versions were synthesised to form one common Mandarin version, T-12.

#### Back-translation

Two native English speakers with Chinese as their second-language (BT-1, BT-2) carried out a back translation of the Mandarin version (T-12) into English. Neither of the back-translators was familiar with the subject matter of the scale; both were blind to the English original and each carried out the translation independently. A third person (a native English clinical research scientist) compared the two back-translations with each other and with the original questionnaire and highlighted any conceptual errors or gross inconsistencies in the content of the translated versions, in preparation for the expert committee meeting.

#### Expert committees meeting

An expert committee was formed consisting of all translators, a scaling methodologist and an assistant researcher with a master’s degree in English translation and a native English clinical research scientist. A committee meeting was organized on November 9, 2017, at which committee members examined the translations, the back translations, and the notes made in carrying out/comparing the translations, and consolidated these to produce a “prefinal” version of the Mandarin sf-DCS. The task of this expert committee was to assure semantic, idiomatic, experiential and conceptual equivalence between the Mandarin and English versions of the sf-DCS. For all parts of the questionnaire (instructions, items, and response options) consensus was eventually reached among committee members. All stages of the translation process, and any discrepancies, problems, or difficulties encountered, were documented in written form. Then, all the documented materials together with the produced “prefinal” version of the Mandarin sf-DCS were emailed to the other people who had been involved in the process of translation, synthesis and back-translation.

#### Methods for improving the colloquial and linguistic aspects of the scale

According to the DCS user manual, respondents with limited response skills find it harder to respond to the statement format than the question version of the scale. To address this issue two ten-year-old Chinese girls at the fourth grade were asked to test the indicative and colloquial aspects of the language used in the scale. The rationale is that through the test we were assured people of 4th grade education level and higher would be able to read and comprehend the scale with no difficulty. They were required to read each item, explain the meaning, state whether they had difficulty in understanding and offer suggestions for modification. Any difficulty in understanding was dealt with by giving paraphrases and replacements of originals, and any suggestion for modification was recorded and considered. If no difficulty of understanding or suggestion for modification arose, it was considered that the items were easy to understand and respond to. The findings from this phase of the adaptation process were evaluated before the final Mandarin version of the sf-DCS was produced and subjected to further psychometric testing.

### Assessment of the psychometric properties of the sf-DCS

#### Questionnaires and samples

Assessment of the psychometric properties of the sf-DCS was conducted in both influenza immunization and breast cancer screening decision-making contexts by online questionnaires. The questionnaire consisted of four parts. The first part collected demographic information. The second part was to present basic and practical health information (information about influenza immunization was presented in the influenza immunization decision-making context and information about breast cancer screening was presented in the breast cancer screening context), and respondents were told that the presented information could be read selectively by themselves. This presumed that, in reality, respondents would read what they need on their own initiative, which may be influenced by many factors including personal interest or need. Then respondents were asked how much of the information provided above they had read, and the response options could be “all of the provided information”, “a part of the provided information” and “none of the provided information”. The third part asked one question that “Assuming that you have sufficient spare time tomorrow and you now need to decide whether or not to receive influenza immunization (in the influenza immunization decision-making context)/ breast cancer screening (in the breast cancer screening decision-making context) tomorrow, which option do you choose?” Response to the question was classified as accept (I choose to receive influenza immunization/ breast cancer screening), reject (I choose not to receive influenza immunization/ breast cancer screening), or unsure (I am unsure about which option to choose), and both the “reject” and “accept” answers were defined as being “sure” about a decision. In the fourth part, respondents were asked to answer the questions of the sf-DCS (Mandarin version). The online questionnaire was open to all people who had access to a popular professional platform for online questionnaires in China. The inclusion criteria was: 1) above 18 years old; 2) having no reading disorders; 3) being willing to be investigated; and 4) being female (only for breast cancer screening decision making investigating). The survey lasted for one month. The number of respondents was calculated as 5~10 patients for each item of the sf-DCS [13], which means between 80~160 respondents for each survey would be acceptable.

#### Statistical analysis

All data analyses procedures were undertaken using SPSS, version 17.0 (SPSS, Inc., Chicago, IL). *P*-values of less than 0.05 were taken to be statistically significant.

To determine the ability of the sf-DCS to discriminate between respondents in terms of whether or not they were unsure about their decision, Mann-Whitney Test was used to compare sf-DCS scores between groups whose decisions or intentions indicated they were “sure (receive/reject)” and “unsure” about the decision to be screened or immunized. According to the decisional conflict construct, individuals who were “unsure” were expected to have significantly higher decisional conflict scores than the other groups.

In this study, factor analysis with varimax rotation was performed on the combined data-set of the two samples to estimate which model would best fit our data, and we canceled items where factor loadings were lower than 0.50 on all factors. The original sf-DCS developed in 1995 [1] was composed of three subscales, and subsequently, the subscale “factors contributing to the uncertainty” was split into three subscales leading to a total of five subscales for the sf-DCS. According to other previous studies [1–4], the sf-DCS’s factor structure is still lacking in clarity and further analysis is warranted. Therefore, in this study, five factor analysis methods were used to test the sf-DCS’s factorial validity. Exploratory factor analysis (EFA) with varimax rotation was conducted to probe the potential structure including five-factor model (varimax rotation) and three-factor model (varimax rotation).

It has been proved that, by providing relevant health information to aid patients decisions, PDAs decreased decision conflict (score of the sf-DCS) [2]. Because we thought that maybe people’s decision conflict was not only correlated to knowledge they had just viewed, but more importantly was also influenced by their prior knowledge or any factor influencing their prior knowledge and understanding of the provided health related information including medical-related occupation and education level. Those who had personal history of chronic diseases, who were older and had a family member with breast cancer might have had more experience or training effects in making a medical related decision which might decrease their decisional conflict in this study. Respondent’s self-reported family economic status might also influence decisional conflict because people have to consider cost when they make a medical decision. Above all, it was assumed that those who had read more of the provided information, had higher self-perceived prior knowledge level, worked in medical related occupations, were older, reported better family economic status, had chronic diseases or had a family member with breast cancer might get lower sf-DCS scores. Therefore, a stepwise linear regression analysis with a total score of the sf-DCS as a dependent variable, and those factors above as independent variables was conducted to explore factors influencing total sores of the sf-DCS in influenza immunization and breast cancer screening decision contexts respectively. The variance inflation factor (VIF) was used to identify correlation between independent variables and the strength of that correlation. A value of 1 indicates that there is no correlation between this independent variable and any others, and VIFs between 1 and 5 suggest that there is a moderate correlation.

In order to assess the test-retest reliability a group of 18–23 year old students (*n* = 30) were selected from one class of a university. They filled in an online questionnaire separately set, and this is a completely separate sample and was retested two weeks later. Test-retest reliability of the scale was evaluated by correlating the test and retest scores using Pearson r.

Reliability of the whole scale and each of the subscales was estimated by calculating Cronbach’s Alpha coefficients, and was investigated separately in the two decision-making contexts as well as in the combination of the two decision-making contexts. Cronbach alpha coefficient for each item was determined by observing the effect of an item’s removal on the overall alpha coefficient of the sf-DCS. An alpha value greater than 0.70 was considered acceptable [14]. Item-to-total correlations were also calculated to assess the internal consistency of the sf-DCS.

## Results

### Cross-cultural adaptation of the sf-DCS

There was no difficulty during its translation and back-translation. However, during the test of the pre-final version, the two ten years old girls could not understand the Chinese translation of “informed choice”, therefore we glossed “informed choice” with an explanation “means you fully understand” in brackets. The two ten years old girls also suggested that the Chinese translation of “I feel sure about what to choose” changed to “I know what I want to choose”. After discussion, we accepted the two girls’ suggestions, because it reads more like Chinese colloquialisms.

### Assessment of the psychometric properties of the sf-DCS

#### Characteristics of respondents (Table 1)

There were 437 respondents in the influenza immunization decision-making context and 238 respondents in the breast cancer screening decision-making context. Demographics and characteristics of all the respondents in the two decision-making contexts are displayed in Table 1. Respondents’ self-perceived prior knowledge of and attitude towards influenza immunization/ breast cancer screening are shown in Table 2.

**Table 1:** Demographic characteristics of the respondents

Demographic characteristics		Decision context	
		Influenza immunization	Breast cancer screening
Age	Mean, SD, range	28.69, 8.732, 18~67	28.56, 10.815, 18~99
Gender	Male	25.2% (110)	0% (0)
	Female	74.8% (327)	100% (238)
Education background	Lower than university	5.5% (24)	8.4% (20)
	University or higher	94.5% (413)	91.6% (218)
Do you have a chronic disease?	No	91.8% (401)	89.1% (212)
	Yes	8.2% (36)	10.9% (26)
Occupation	Medical related occupation	40.7% (178)	45.4% (108)
	Non-medical related occupation	24.7% (108)	16.0% (38)
	Not in work (students or unemployed)	34.6% (151)	38.7% (92)
Self-reported family economic status	Very poor	8.0%(35)	4.6%(11)
	Poor	9.2%(40)	10.1%(24)
	Ordinary	76.2%(333)	77.7%(185)
	Good	6.4%(28)	7.1%(17)
	Very good	0.2%(1)	0.4%(1)
Do you have a family member with breast cancer?	No	–	89.5 (213)
	Yes	–	10.5% (25)

*SD* standard deviation

**Table 2:** Self-perceived prior knowledge of and attitude towards influenza immunization/breast cancer screening

Characteristics		Decision context	
		Influenza immunization	Breast cancer screening
Self-perceived prior knowledge level of influenza immunization/breast cancer screening	Full knowledge	5.7%(25)	5.9%(14)
	Partial knowledge	83.3%(364)	76.1%(181)
	No knowledge	11.0%(48)	18.1%(43)
Have you read the information provided about influenza immunization/breast cancer?	Have read all the information	62.5%(273)	60.5%(144)
	Have read a part of the information	32.0%(140)	36.1%(86)
	Haven’t read the information at all	5.5%(24)	3.4%(8)
Assuming that you have enough spare time tomorrow and now you need to decide whether to receive influenza immunization/ breast cancer screening or not, which option do you choose?	Accept	44.2%(193)	45.4%(108)
	Reject	34.5%(151)	34.4%(82)
	Unsure	21.3%(93)	20.2%(48)

*SD* standard deviation

#### Criteria validity of the sf-DCS

The criterion validity was estimated in both samples separately (see Table 3). It was suggested that the sf-DCS was consistent in discriminating significantly between those who were “sure” and “unsure” of the decision to be immunized/screened (*P* < 0.05). Higher scores, indicating higher decisional conflict, were obtained among respondents who were “unsure” about whether to be immunized or screened. Similar patterns were observed with all the subscales.

**Table 3:** Difference between groups who are “sure” and “unsure” of a decision to be immunized or screened for breast cancer

Decision context	Scale	Mean; SD (p25; p75)	Decision/Intention		*P* value	Z value
			Sure: Mean; SD	Unsure: Mean; SD		
Influenza immunization	Total	26.3; 15.7 (20.3; 34.4)	24.3; 15.9	33.7; 12.5	< 0.001	−5.988
	Informed	28.1; 17.8 (16.7; 41.7)	25.8; 18.1	36.3; 13.4	< 0.001	−5.887
	Values clarity	25.3; 16.6 (16.7; 33.3)	23.5; 16.6	31.9; 14.8	< 0.001	−4.670
	Support	29.5; 19.1 (20.8; 41.7)	27.5; 19.4	36.6; 15.7	< 0.001	−4.763
	uncertainty	26.5; 18.7 (16.7; 33.3)	24.2; 18.6	35.0; 16.2	< 0.001	−5.542
	Effective decision	23.4; 15.9 (12.5; 25.0)	21.7; 16.0	29.9; 14.1	< 0.001	−4.804
Breast cancer screening	Total	26.5; 14.8 (21.5; 34.4)	25.1; 13.9	32.1; 16.9	0.003	−2.940
	Informed	27.1; 16.7 (25.0; 33.3)	25.8; 16.6	31.9; 16.4	0.006	− 2.755
	Values clarity	24.2; 14.7 (16.7; 27.1)	22.8; 13.5	30.0; 17.8	0.007	−2.684
	Support	29.4; 17.4 (25.0; 41.7)	28.2; 16.9	33.9; 18.8	0.034	−2.123
	uncertainty	27.2; 18.0 (25.0; 33.3)	25.4; 17.1	34.0; 19.7	0.001	−3.248
	Effective decision	25.1; 15.1 (18.8; 31.3)	23.6; 14.2	31.0; 17.1	0.001	−3.189

SD: standard deviation, p25: the first quartile, p75: the third quartile; P value: approximate values to three decimal place

#### Factorial validity of the sf-DCS

The results of the EFA showed that the questionnaire was appropriate for factor analysis (Kaiser-Meyer-Olkin was 0.959, the chi-square value of Bartlett’s test was 10,338.108, and the significance was < 0.001), and factors with Eigen value equaling to 1 were extracted. However, only one factor was extracted and the axis could not be rotated. The factor loading of the revised five-factor model (varimax rotation) and three-factor model (varimax rotation) are shown in Table 4. Several items loaded onto more than one factor. The item “sf-DCS 8” was removed in the revised five-factor model (varimax rotation) and three-factor model (varimax rotation) because it failed to load onto the expected factor. The items “sf-DCS 1” and “sf-DCS 6” were also removed in the revised five-factor model (varimax rotation) because they failed to load onto the expected factor. Besides, the item “sf-DCS 6” had cross-loadings both in the five-factor model (varimax rotation) and three-factor model (varimax rotation). Therefore, it was found that the item “sf-DCS 8” was distinct from the item “sf-DCS 7” and “sf-DCS 9” both in the revised five-factor and three-factor models, and simultaneously the item “sf-DCS7” and “sf-DCS 8” were together forming an independent factor.

**Table 4:** Factor structure of the sf-DCS (principal component analysis, *n* = 675)

Items	One factor extracted	Five factors					Three factors		
		1	2	3	4	5	1	2	3
sf-DCS 1: I know which options are available to me.	0.744	-	-	-	-	-	0.663		
sf-DCS 2: I know the benefits of each option.	0.813	0.750					0.719		
sf-DCS 3: I know the possible risks and side effects of each option.	0.794	0.742					0.768		
sf-DCS 4: I am clear about which benefits matter most to me.	0.796		0.782				0.755		
sf-DCS 5: I am clear about which risks and side effects matter most to me.	0.810		0.723				0.761		
sf-DCS 6: I am clear about which is more important to me (the benefits or the risks and side effects).	0.810	-	-	-	-	-	-	-	-
sf-DCS 7: I have enough support from others to make a choice.	0.732			0.820				0.816	
sf-DCS 8: I am choosing without pressure from others.	0.680	-	-	-	-	-	-	-	-
sf-DCS 9: I have enough advice to make a choice.	0.759			0.744				0.779	
sf-DCS 10: I am clear about the best choice for me.	0.880				0.695				0.654
sf-DCS 11: I feel sure about what to choose.	0.884				0.738				0.671
sf-DCS 12: This decision is easy for me to make.	0.834				0.703				0.665
sf-DCS 13: I feel I have made an informed choice.	0.860					0.532			0.688
sf-DCS 14: My decision shows what is important to me.	0.852					0.736			0.802
sf-DCS 15: I expect to stick with my decision.	0.831					0.837			0.843
sf-DCS 16: I am satisfied with my decision.	0.858					0.799			0.819

Factor loadings between −0.50 and 0.50 are omitted

#### Factors influencing the sf-DCS

As shown in Table 5, in both the influenza immunization decision context and breast cancer screening decision context, respondents who were “unsure” scored higher on sf-DCS compared with people who were “sure” about their decisions/intentions related to influenza immunization/breast cancer screening. Similarly, respondents who had read less information or reported lower self-perceived prior knowledge level had higher total sores. In the breast cancer screening decision context, respondents who were not in work got higher sf-DCS scores compared with respondents in non-medical related occupations. In the influenza immunization decision context, the sf-DCS scores decreased with the increase of respondents’ age.

**Table 5:** Factors influencing total scores of the sf-DCS in influenza immunization decision context and breast cancer screening decision context

Decision context	Independent variable	Standardized coefficients	Standard error	*P* value	t	Variance inflation factor (VIF)
Influenza immunization decision context (*n* = 437)	Self-perceived prior knowledge level	0.209	1.741	< 0.001	4.661	1.052
	Decisions/ intentions	0.217	1.692	< 0.001	4.928	1.013
	Have you read the information provided about influenza immunization?	0.201	1.172	< 0.001	4.521	1.029
	Age	−0.089	0.079	0.043	−2.032	1.003
Breast cancer screening decision context (*n* = 238)	Self-perceived prior knowledge level	0.245	1.895	< 0.001	4.033	1.011
	Decisions/intentions	0.177	2.245	0.004	2.902	1.020
	Occupation (“Not in work” compared to “non-medical related occupation”)	0.160	1.866	0.009	2.621	1.022
	Have you read the information provided about breast cancer screening?	0.131	1.627	0.034	2.134	1.029

#### Reliability of the sf-DCS

There was no statistically significant difference between test and retest scores. The test-retest correlation coefficient after two weeks was 0.528.

Cronbach’s Alpha coefficients calculated on the total scale and each of the subscales are presented in Table 6, and item-to-total correlations and Cronbach’s Alpha for total scale after the item has been deleted ranged from 0.959 to 0.965.

**Table 6:** Cronbach’s coefficients on the total scale and subscales

Scale	Influenza immunization (*n* = 437)	Breast cancer screening (*n* = 238)	Overall (*n* = 675)
Total scale	0.936	0.965	0.963
Informed	0.847	0.883	0.858
Values clarity	0.880	0.889	0.882
Support	0.800	0.784	0.793
Uncertainty	0.929	0.925	0.927
Effective decision	0.937	0.930	0.935

## Discussion

### Criteria validity

The results suggest that the difference between the “sure” and “unsure” were significant, which was consistent with the original study by O’Connor [1] and other language versions of the sf-DCS development studies including the Dutch [9] and French [10] versions. The demonstrated capability of the sf-DCS to discriminate between groups “sure” and “unsure” confirmed that the Mandarin sf-DCS has satisfactory criterion validity.

### Factorial validity

In our study, the revised five factor model was the one with best fit. Three items were removed in this revised model. The item “sf-DCS 8” was removed because it failed to fit onto the expected factor, which was consistent with a previous study [15]. In practice the reason that the item “sf-DCS 8” was removed might be because the “pressure from others” was too vague for respondents. There was no explanation or examples of which kind of pressure, nor who the “others” refer to, which could make this item unclear. The item “sf-DCS 6: I am clear about which is more important to me (the benefits or the risks and side effects)” involved a deep analysis in the decision process, which means respondents must have synthesized all benefits and all the risks and then compare them in total. However, as humans, we each possess a fast and impression-based system of decision-making, in addition to a slower, reflective one capable of complex calculations that checks and verifies our quick impressions [16–18], except in those individuals who have practiced significant exercise of thoughts and intelligence. In this study, respondents were asked to make a choice and then were given the sf-DCS to respond to. Therefore we believe the item “sf-DCS 1: I know which options are available to me” may be a redundant item for respondents because conflict is a property of the choice situation [6].The item implies that decisional conflict emerged after respondents had already known available options and thought about the decision. Perhaps there is no need to ask respondents if they know available options after they had contemplated on a decision.

### Factors influencing the sf-DCS

In the two decision making contexts, it was suggested that compared with “sure” group, the “unsure” group had got higher sf-DCS scores, which confirmed sf-DCS’s criterion validity. Regarding the influence of provided health related information and respondent’s self-perceived prior knowledge, the results were consistent with our assumptions. In the influenza immunization decision context, the influence of age on sf-DCS scores was consistent with our assumption, but not in the breast cancer screening context, which might be because the morbidity of breast cancer was correlated with age. In the breast cancer screening decision context, respondents who were not in work got higher sf-DCS scores, which was inconsistent with our assumption and needs further study.

### Reliability

All the other language versions of the sf-DCS development studies reported a satisfactory internal consistency of subscales and total scales of the sf-DCS [8–11], which was consistent with the results of this study. Cronbach’s Alpha coefficients calculated on the total scale for the sf-DCS in the two decision-making contexts separately as well as in the two decision-making contexts together were high, and Cronbach’s Alpha coefficients calculated on the five individual subscales for the sf-DCS were all acceptable. However, Cronbach’s Alpha of total scale after a specific item has been deleted suggested that the Cronbach’s Alpha of the item “sf-DCS 8” was the lowest among all the 16 items.

In this study, the test-retest results of the sf-DCS were not satisfactory. None of the previous studies reported test-retest reliability. Because, decisional conflict has been defined as status of uncertainty it is reasonable to think this status could change over time due to many variables including personal experience and the influence of outside information. Therefore, the test-retest reliability may not be suitable for decision processes.

## Limitations

First, our online surveys are open to all people who have access to a popular professional survey platform in China. The distribution of the surveyed sample might represent the natural distribution of our netizens. Although the internet has been the primary way a lot of people access information, some older people and those with a very low education level may not have acquired the skills of accessing to online information and would therefore be very likely to miss our questionnaire. For this reason, the influence of respondent’s age and education level on the sf-DCS scores, as reported in our study, should be considered prudently. Second, the respondents were asked to imagine a situation in which they were facing the decision making context, which might be difficult to perceive as realistic. Although, using hypothetical situations of medical decision making is effective in assisting individuals to understand the decision making process [18, 19]. Third, our study did not incorporate family and friends involvement in decision making, which might have enabled the respondents to relate their answers to a variety of medical decisions or intentions. This might influence respondent’s understanding and perceptions of the item “sf-DCS 8”, because respondent’s decision might be influenced by their family members or friends.

## Conclusions

In conclusion, the Mandarin version of the sf-DCS had confirmed good criteria validity and a fitted revised five factors model by removing three items, and maybe the sf-DCS’ factor structure needs to be refined based on a clearer and rethought theoretical framework by synthesizing the most current research conclusions. Although the test-retest reliability was not satisfactory; the internal consistency of the sf-DCS was good. Overall, our study suggests that the Mandarin version of sf-DCS is valid to be used in mainland China, but caution should be taken when using any of its subscales separately to assess the patient decision-making process.

## Supplementary information

**Additional file 1.** The statement format Decisional Conflict Scale.

## Data Availability

The datasets used and/or analyzed during the current study are available from the corresponding author on reasonable request.

## Abbreviations

DCS: Decisional Conflict Scale
EFA: Exploratory factor analysis
sf-DCS: Statement format Decisional Conflict Scale
VIF: Variance inflation factor
