# Supplementary material for: Cross-cultural adaptation and psychometric assessment of the statement format Decisional Conflict Scale for Mandarin version
Source: BMC Health Serv Res. 2019 Nov 21;19:873. doi: 10.1186/s12913-019-4717-6 (PMC6873697; doi:10.1186/s12913-019-4717-6)
Supplement: Supplementary file 1 — Additional file 1. The statement format Decisional Conflict Scale. [file 12913_2019_4717_MOESM1_ESM.doc]

Appendix: the original Decisional Conflict Scale (Statement Format)

A. Which [insert treatment/ screening] option do you prefer? Please check one.

 [Option 1]

 [Option 1]

 Unsure

B. Considering the option you prefer, please answer the following questions:

|  | Strongly agree | Agree | Neither agree nor disagree | Disagree | Strongly disagree |
| --- | --- | --- | --- | --- | --- |
| 1. I know which options are available to me. |  |  |  |  |  |
| 1. I know the benefits of each option. |  |  |  |  |  |
| 1. I know the possible risks and side effects of each option. |  |  |  |  |  |
| 1. I am clear about which benefits matter most to me. |  |  |  |  |  |
| 1. I am clear about which risks and side effects matter most to me. |  |  |  |  |  |
| 1. I am clear about which is more important to me (the benefits or the risks and side effects). |  |  |  |  |  |
| 1. I have enough support from others to make a choice. |  |  |  |  |  |
| 1. I am choosing without pressure from others. |  |  |  |  |  |
| 1. I have enough advice to make a choice. |  |  |  |  |  |
| 1. I am clear about the best choice to me. |  |  |  |  |  |
| 1. I feel sure about what to choose. |  |  |  |  |  |
| 1. This decision is easy for me to make. |  |  |  |  |  |
| 1. I feel I have made an informed choice. |  |  |  |  |  |
| 1. My decision shows what is important to me. |  |  |  |  |  |
| 1. I expect to stick with my decision. |  |  |  |  |  |
| 1. I am satisfied with my decision. |  |  |  |  |  |
